# Research progress of MUC1 in genitourinary cancers

**DOI:** 10.1186/s11658-024-00654-x

**Published:** 2024-11-03

**Authors:** Weipu Mao, Houliang Zhang, Keyi Wang, Jiang Geng, Jianping Wu

**Affiliations:** 1 Department of Urology, Affiliated Zhongda Hospital of Southeast University, No. 87 Dingjiaqiao, Gulou District, Nanjing, 210009 Jiangsu China; 2 Department of Urology, Zhongshan Hospital, Fudan University, Shanghai, People’s Republic of China; 3 Department of Urology, Bengbu First People’s Hospital, Bengbu, People’s Republic of China; 4 Department of Urology, Shanghai Tenth People’s Hospital, Tongji University, Shanghai, People’s Republic of China

**Keywords:** MUC1, Genitourinary cancers, Diagnosis, Therapeutic target

## Abstract

*MUC1* is a highly glycosylated transmembrane protein with a high molecular weight. It plays a role in lubricating and protecting mucosal epithelium, participates in epithelial cell renewal and differentiation, and regulates cell adhesion, signal transduction, and immune response. *MUC1* is expressed in both normal and malignant epithelial cells, and plays an important role in the diagnosis, prognosis prediction and clinical monitoring of a variety of tumors and is expected to be a new therapeutic target. This article reviews the structural features, expression regulation mechanism, and research progress of *MUC1* in the development of genitourinary cancers and its clinical applications.

## Background

Mucins are a family of highly glycosylated proteins that are mainly expressed at the apical border of epithelial cells, and their extracellular domains contain variable number of tandem repeat sequences (VNTRs) consisting of abundant proline, threonine, and serine (PTS), which distinguish them from other glycoproteins [1]. Based on their functions, mucins can be classified into membrane-bound and secreted types. Mucin1 is a member of the membrane-bound mucin family, characterized as a single-pass type I transmembrane glycoprotein that is highly *O*-glycosylated and contains contiguous VNTRs, making it a high molecular weight glycoprotein [2, 3]. Under normal circumstances, *MUC1* is primarily expressed in the apical surfaces of epithelial cells in various tissues and organs, where it serves as a protector and lubricant [4].

As a member of membrane-bound mucins, *MUC1* can cross cell membranes and act as an adhesion molecule for cancer cells, contributing to extravascular metastasis of cancer cells and mediating intercellular signal transduction and adhesion. Aberrant expression of in tumor tissues is closely related to tumorigenesis and progression, exhibiting a high positive rate in tumor marker detection [5, 6]. Therefore, *MUC1* is expected to become a novel marker indicating tumor progression, holding significant value in clinical diagnosis, treatment and prognosis assessment of cancer. Moreover, numerous studies have shown that *MUC1* is an oncogene, making it an attractive target for cancer immunotherapy [7]. Since *MUC1* is primarily expressed in epithelial cells, common cancers of the urinary system, such as bladder cancer (BCa), renal cell carcinoma (RCC), and prostate cancer (PCa), all originate from epithelial cells. Studies show that *MUC1* is abnormally overexpressed in PCa, BCa, and RCC, and plays an important role in tumor progression [8]. This article reviews the structure and function of *MUC1*, the mechanism of expression regulation, as well as research progress and clinical application of *MUC1* in the development of urological cancers, hoping to provide new insights for the treatment of urological cancers (see Table 1).

**Table 1:** MUC1-based clinical trials

Cancer type	Biological	Composition and targets	Study phase	Trial number	Status	Year	Enrollment
Bladder cancer	PANVAC	CEA-MUC1-TRICOM	II	NCT02015104	Completed	2013	32
	CV301	CEA, MUC1, ICAM-1, LFA-3, and B7-1	II	NCT03628716	Halted	2018	43
	Dendritic cells	MUC1	II	NCT04184232	Completed	2019	17
Renal cell carcinoma	Vaccine	MUC1, CEA, Her2/neu, telomerase, survivin, MAGE-A1	I/II		Completed	2003	30
	TG4010	MUC1-IL2	II		Completed	2003	37
	Antibody	Anti-CD3-MUC1	II	NCT03540199	Withdrawn	2018	0
	Dendritic cells	MUC1-PADRE	I/II		Completed	2020	20
	CAR-T cells	P-MUC1C-ALLO1	I	NCT05239143	Recruiting	2022	100
Prostate cancer	Vaccine	VV/MUC-1/IL-2	I		Completed	1998	22
	Vaccine	MUC1-KLH	I	NCT00005632	Completed	1999	27
	TG4010	MUC1-IL2	II	NCT00040170	Terminated	2002	40
	Vaccine	MUC1	I	NCT00374049	Completed	2006	14
	Dendritic cells	Tn-MUC1	I/II	NCT00852007	Completed	2009	20
	L-BLP25	MUC1	II	NCT01496131	Completed	2011	28
	CV9104	PSA, PSMA, PSCA, STEAP, PAP, and MUC1	I/II	NCT01817738	Terminated	2012	197
	Vaccine	Ad-sig-hMUC1/ecdCD40L	I	NCT02140996	Unknown	2014	24
	CV9104	PSA, PSMA, PSCA, STEAP, PAP, and MUC1	II	NCT02140138	Terminated	2014	35
	Dendritic cells	NY-ESO-1, MAGE-C2, and MUC1	II	NCT02692976	Completed	2015	21
	Vaccine	Ad5 CEA/MUC1/Brachyury	I	NCT03384316	Completed	2018	11
	Vaccine	Ad5 PSA/MUC1/brachyury	I	NCT03481816	Completed	2018	18

## MUC1 structure and function

### MUC1 structure

**Fig. 1:**
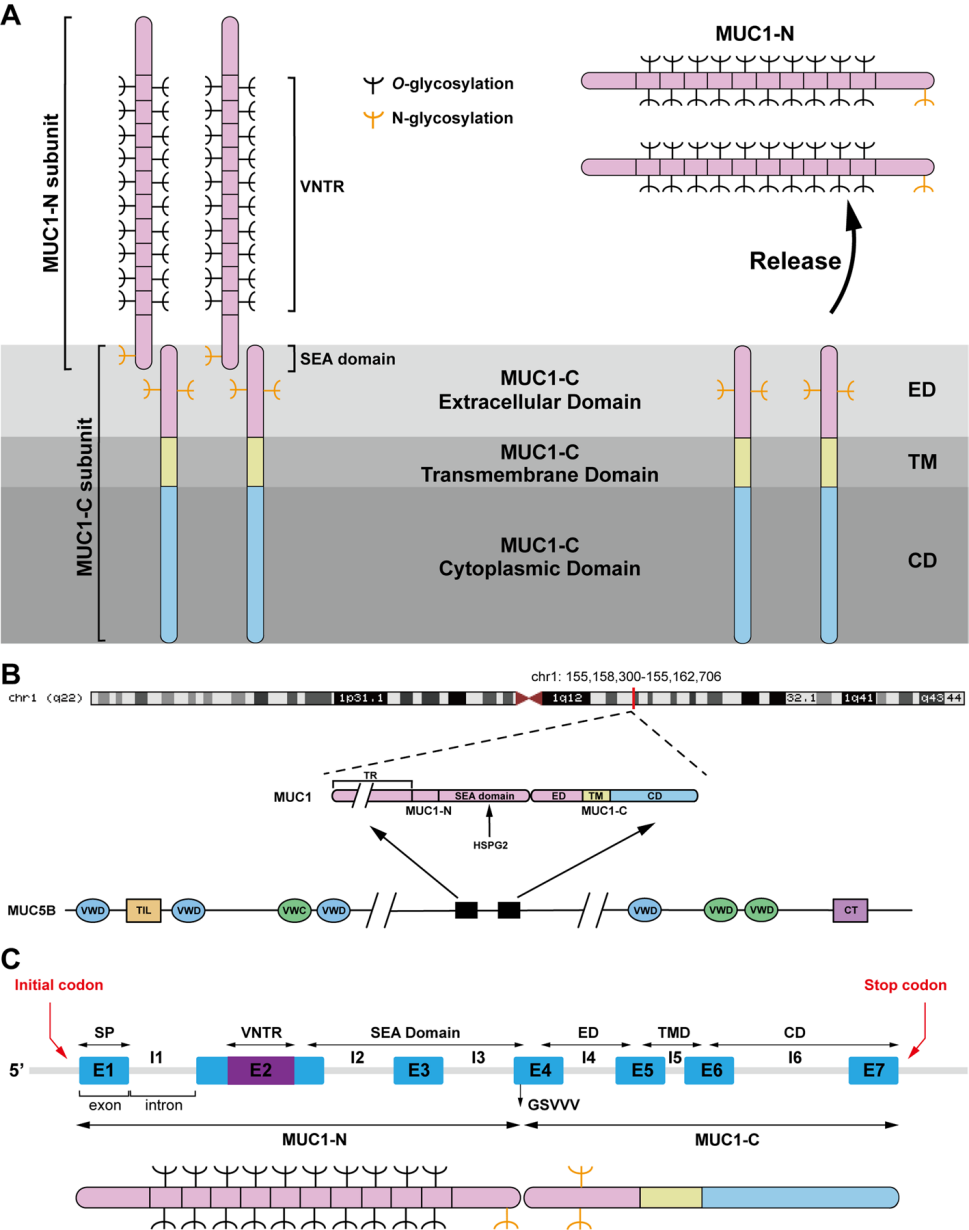
Structure of the *MUC1* gene encoding the MUC1-N and MUC1-C subunits. **A** *MUC1* sequences emerged from *MUC5B*. **B** Schematic representation of the MUC1-C gene, which consists of 7 exons (E1–E7) and 6 introns (I1–I6). MUC1-N is encoded by exons 1–4 and MUC1-C encoded by exons 4–7. **C** Schematic representation of the MUC1-C gene, which consists of seven exons (E1–E7) and six introns (I1–I6). MUC1-N is encoded by exons 1–4 and MUC1-C is encoded by exons 4–7. Structure of MUC1, consisting of extracellular domain (ED), transmembrane domain (TM), and cytoplasmic domain (CD). MUC1 releases MUC1-N into the protective physical barrier in response to inflammation

*MUC1* is a polymorphic transmembrane glycoprotein with high glycosylation and is composed of a core peptide and sugar chains, with sugar chains accounting for 50–90% of its mass, and the core peptide consists of an extracellular domain (ED), transmembrane domain (TM), and cytoplasmic domain (CD) [9]. The ED determines the speciffic spatial structure and immunogenicity of *MUC1*, and the TM and CD are highly conserved among different species, which may be related to its tissue-specific expression [4, 10]. When normal cells undergo carcinogenesis, they lose polarity and release the *MUC1* extracellular segment (Fig. 1A) [1].

*MUC1*, which is localized to 1q22, evolved in part from the gene that encodes the mucin 5B (*MUC5B*) secreted mucin (Fig. 1B) [11, 12]. *MUC1* consists of seven exons (Fig. 1C). The *MUC1* protein is expressed as a single polypeptide that undergoes autocleavage at a sea urchin spermatidin, enterokinase, and agarose (SEA) domain into MUC1 N-terminal (MUC1-N) and C-terminal (MUC1-C) subunits (Fig. 1C). The *MUC1* polypeptide is hydrolyzed post-translationally by autoproteins at the GSVVV motif of the SEA domains. In turn, extracellular MUC1-N (KQGGFLGLSNIKFRPG) and transmembrane MUC1-C (SVVQLTLAFREGTINVHDV) can form heterodimeric complexes through stable hydrogen bonding, transported from the endoplasmic reticulum (ER) to the golgi apparatus for glycosylation modification, and then localized to epithelial cell membranes, resulting in the expression of the MUC1-N/MUC1-C non-covalent complex on the cell surface [13–16].

#### MUC1-N subunit

MUC1-N is encoded by exons 1–4 and contains the core portion of mucin, includes 20–120 VNTRs and SEA structural domains [17, 18]. The amino acid sequence of the VNTRs domain varies in different tumor cell lines. The VNTRs consist of a 20-amino acid motif (PDTRPAPGSTAPPAHGGVTSA) and are within the sequence rich in proline, serine, and threonine, also known as the PTS domain [19]. Proline imparts a rigid, inflexible structure to the *MUC1* molecule, while serine and threonine serve as *O*-glycosylation sites [20].

#### MUC1-C subunit

**Fig. 2:**
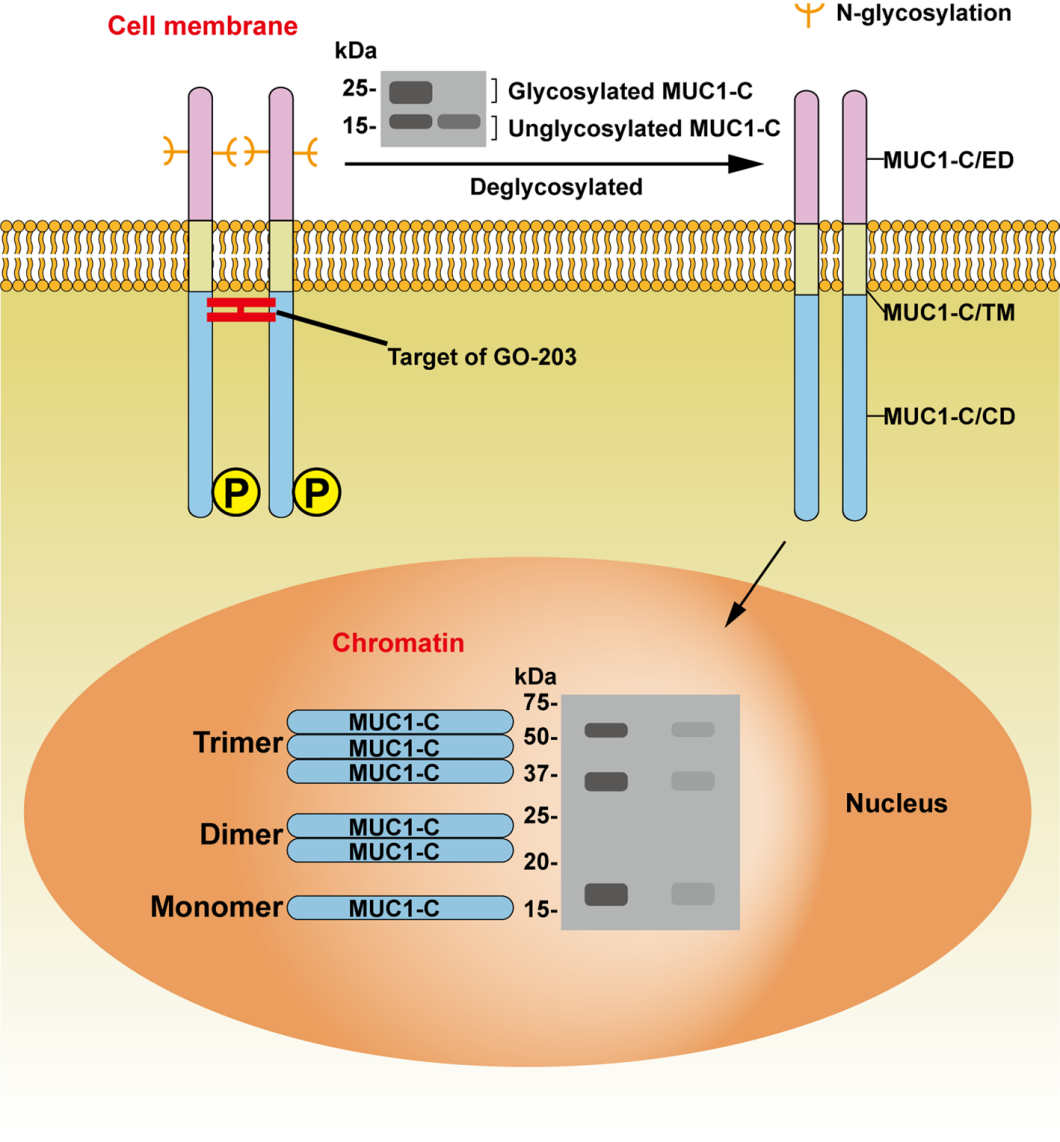
Localization of MUC1-C to chromatin. Unglycosylated MUC1-C has a molecular weight of 17 kDa and increases up to 25 kDa with increasing MUC1-C/CD *N*-glycosylation. MUC1-C exists as a 17 kDa monomer and a 34 kDa homodimer in chromatin

MUC1-C, encoded by exons 4–7, is highly conserved in mammals and consists of a 58 amino acids (aa) ED, a 28 aa TM, and a 72 aa CD that anchors MUC1-N to the cell surface [21, 22]. Unglycosylated MUC1-C has a molecular weight of 17 kDa, and with increasing *N*-glycosylation of MUC1-C/CD, the molecular weight can reach 25 kDa [13]. Recently, our research revealed that MUC1-C is localized in chromatin, the MUC1-C 17 kDa protein is localized in chromatin as a 17 kDa monomer and a 34 kDa homodimer, and MUC1-C may also exist in a homotrimeric form (Fig. 2) [23]. The cell-penetrating peptide GO-203 can directly bind to the CQC motif of MUC1-C/CD, effectively blocking the reactivity of this site, thereby inhibiting MUC1-C function [24].

### MUC1 function

Under normal circumstances, *MUC1* is primarily expressed in epithelial cells and their secreted mucus, widely distributed in the epithelial cells of the human respiratory tract, mammary glands, gastrointestinal tract, and genitourinary (GU) tract [25]. It plays a crucial role in mucosal protection, lubrication, signaling transduction, and local natural immunity [1, 26].

The MUC1-N ranges from 200 to 500 nm in length, with antigenic epitopes concentrated mainly in the domain of VNTRs, which is one of the membrane surface molecules first encountered by the body’s immune system, and many monoclonal antibodies react with *MUC1* at these sites [14, 27]. MUC1-N is also *N*-glycosylated in the domain close to the cell membrane, aiding in its secretion, localization, and folding [28]. The PDTRP site in the VNTRs domain is a common epitope recognized by B cells and T cells, enabling *MUC1* to participate in cellular immunity [29]. The fully glycosylated MUC1-N subunit extends above the glycocalyx to form a physical barrier that can produce a lubricating effect on mucosal surfaces and protects cells from external physicochemical factors (infections, toxins, physical damage, and other forms of stress) [30–32].

MUC1-C is capable of being phosphorylated by a variety of kinases, allowing MUC1-C to integrate inflammatory and proliferative responses [33]. MUC1-C has the ability to activate stem cell functions for repair and remodeling of the barrier regeneration. MUC1-C is activated in the response of the barrier tissues to stress, inducing inflammatory, proliferative, and remodeling pathways associated with wound healing and repair [34]. In addition, MUC1-C can interact with specific proteins involved in tumor proliferation, angiogenesis, drug resistance, and immune escape [23, 35, 36]. In addition, the release of MUC1-N is able to activate MUC1-C, inducing epithelial mesenchymal transition (EMT), repair, and reestablishment of homeostasis after inflammation subsides.

## Regulatory mechanisms

### Changes in phosphorylation levels

*MUC1* and its isoforms are expressed on the cell surface and contain ED, TM, and CD. They have a structure similar to various cell surface receptors, suggesting their potential involvement in signaling as membrane surface receptors [37]. Tyrosine (Tyr) phosphorylation is a key step in membrane receptor-mediated signal transduction [38]. Tyr residues on *MUC1* and its CD can be phosphorylated and two potential SH2 domain binding sites and one potential growth factor receptor binding protein 2 (*GRB2*) binding site (pYTNP) [39, 40]. Furthermore, studies found that the amino acid sequences of *MUC1* and its ED have high homology with cell factor receptors such as human growth hormone receptor (*GHR*), human interleukin-7 receptor (*IL-7R*), and mouse interleukin-3 receptor (*IL-3R*), suggesting that *MUC1* might be a class of cytokine receptors [41].

### Changes in glycosylation levels

Glycosylation is one of the essential post-translational modifications of proteins, and changes in glycosylation are required for tumor growth and progression [42, 43]. ED glycosylation is the main post-translational modification process for *MUC1* after translation, and *MUC1* glycosylation is tissue specific and highly polymorphic [20, 44]. MUC1-N contains VNTR sequences, each of which consists of 20 aa residues with abundant *O*-glycosylation sites on serine and threonine residues [20]. The MUC1-N concatenated sequence contains *N*-glycosylation sites, which undergo *O*-glycosylation and *N*-glycosylation in the golgi apparatus and endoplasmic reticulum, respectively [45]. The sugar chains on MUC1-N can stimulate the body to generate major histocompatibility complex (MHC)-mediated immune responses. During the process of antigen recognition and presentation, the *O*-glycan structures of *MUC1* form complexes with MHC molecules and are jointly presented to T cells [46]. When cells undergo malignant transformation, abnormal *O*-glycosylation leads to the conversion of the original *O*-glycan structures into Tn antigens, sialyl Tn antigens, or T antigens, making *MUC1* a tumor antigen recognizable by the immune system [47]. *O*-glycosylation is correlated with the biological properties of *MUC1*, whereas *N*-glycosylation plays an important role in protein folding, secretion, and apical expression [45]. Highly *O*-glycosylated epidermal *MUC1* is a marker for monitoring tumor recurrence [48].

### Adhesion and anti-adhesion effects

Reduced intercellular adhesion in tumor cells and the adhesion of circulating tumor cells to distant organs vascular endothelium are critical steps in tumor metastasis. Adhesion is the first step of tumor cell invasion and metastasis [49, 50]. Tumor cells firstly adhere to the basement membrane and extracellular matrix on fibronectin, laminin and type IV collagen through membrane surface receptors [51]. Subsequently, tumor cells degrade the basement membrane and matrix through proteases, leading to metastasis via the bloodstream and lymphatic system [52]. Studies have shown that in tumor patients, the loss of epithelial cell polarity results in significant underglycosylation, leading to the overexpression of *MUC1* [1, 20]. Overexpressed *MUC1* molecules in cancer cells hinders the interaction between ligands and their receptors on the cell membrane surface, reducing cell–cell interactions mediated by extracellular matrix integrins [53, 54].

E-cadherin, a calcium-dependent cell adhesion molecule, forms a complex with β-catenin in the cytoplasm, mediating cell–cell adhesion and inhibiting tumor cell migration [55]. Downregulation of E-cadherin expression is one of the manifestations of enhanced tumor cell invasiveness. High expression of *MUC1* competitively binds to β-catenin at cell junctions, dissociating the E-cadherin–β-catenin complex and upregulating the expression of EMT inducers, which leads to cytoskeletal destabilization of the intercellular adhesive junction rearrangement, reducing intercellular adhesion between cancer cells and promoting basement membrane invasion [56, 57]. Glycogen synthase kinase 3β (*GSK3β*) can bind to the STDRSPYE sequence of the MUC1-C/CD, phosphorylating serine and decreasing *MUC1* binding to β-catenin, enhancing intercellular adhesion strength [58, 59]. Moreover, *MUC1* can promote the phosphorylation of extracellular signal-regulated kinase 1/2 (*ERK1/2*) and *AKT* by binding to galectin-3 (*GAL3*), enhancing cancer cell adhesion to vascular endothelial cells and tumor invasiveness [60]. Sialyl Lewis x epitope on *MUC1 *serves as a ligand for E-selectin, interacting with E-selectin on damaged or inflamed vascular endothelial cells, promoting adhesion between cancer cells and vascular endothelial cells [61, 62]. This facilitates the passage of cancer cells through the blood vessel wall, thereby aiding in cancer cell metastasis.

### Immunoregulatory role

Cytotoxic T lymphocytes (CTLs) are present in tumor patients and can participate in tumor immunity by killing tumor cells through both MHC-restricted and non-restricted immune response pathways [63, 64]. *MUC1* glycoprotein contains natural IgG antibodies and endogenous anti-*MUC1* antibodies have a protective effect and keep the organism in a tumor-free state through an antibody-mediated host immune surveillance mechanism [65]. *MUC1* is one of the cell surface molecules that the immune system first encounters. It can activate CTLs to kill tumor cells expressing *MUC1*. The PDTRP site in the VNTR domain is recognized by both B cells and T cells [66, 67]. *MUC1* can undergo quantitative and qualitative changes during carcinogenesis, resulting in new antigenic sites. *MUC1* can induce immune response in CTLs while inhibiting the cytotoxic effects of immune active cells on tumor cells [68]. Furthermore, the sTn epitope on the surface of *MUC1* can also inhibit the cytotoxic activity of natural killer (NK) cells [69]. Due to the highly abnormal expression of *MUC1* on the surface of tumor cells, high levels of *MUC1* expression are negatively correlated with the prognosis of cancer patients, suggesting that *MUC1* may be involved in the regulation of immune response and could potentially become a target molecule for cancer immunotherapy [7]. On this basis, various MUC1-based antigens are currently being studied as vaccines for cancer treatment, with some entering clinical trial phases.

### Involved in tumor progression

Tumor development is a complex process involving various events both inside and outside the cell. The expression level of *MUC1* is markedly elevated in malignant tumor cells, which are nonpolarly distributed on the surface and cytoplasm of the epithelial cells [5, 6]. And the appearance of aberrant glycosylation leads to the formation of new glycan epitopes and the exposure of peptide chain epitopes, which cause the tumor cell-specific antigenic epitope formation, making *MUC1* a tumor-associated antigen (TAA) recognized by the immune system [70, 71]. Due to the abnormally high expression of *MUC1* in tumor cells and the loss of cell polarity, *MUC1* is spread over the entire cell surface and can be detected in the blood as small fragments under the hydrolysis of various enzymes [72]. *MUC1* fragments shed into the bloodstream can competitively bind to antibodies injected into the body, thereby affecting the effectiveness of immunotherapy for tumors [73]. Moreover, *MUC1* is involved in the biological processes of tumor cells by regulating proliferation, epithelial–mesenchymal transition, and epigenetics, thus playing a significant role in the occurrence and development of tumors [74]. The high expression of *MUC1* in tumors makes it a potential tumor biomarker and therapeutic target, applied in the diagnosis and biological treatment of various cancers. Therefore, inhibiting the activity of *MUC1* could be an effective approach in cancer treatment.

## MUC1 and GU cancers

### MUC1 and RCC

RCC is the most common malignant kidney tumor in adults, accounting for 3% of adult malignancies. The prognosis of RCC is closely related to the clinical stage, but early diagnosis of RCC is difficult due to the lack of sensitive tumor markers and specific clinical manifestations [75]. For early-stage RCC, surgery is the primary treatment, including partial nephrectomy and radical nephrectomy [76]. Additionally, RCC is resistant to both radiotherapy and chemotherapy, leaving limited effective treatment options for advanced patients. Targeted therapy, a rapidly developing treatment in recent years, focuses on specific molecular pathways in cancer cells and is commonly used for advanced or metastatic RCC [77]. The main targeted therapies include tyrosine kinase inhibitors (TKIs), mammalian target of rapamycin (*mTOR*) inhibitors, and vascular endothelial growth factor (*VEGF*) inhibitors. Immune checkpoint inhibitors have also made significant progress in RCC treatment in recent years, working by blocking immune suppression pathways and enhancing the immune system’s ability to recognize and kill cancer cells, with programmed cell death 1 (*PD-1*)/programmed death-ligand 1 (*PD-L1*) inhibitors and cytotoxic T-lymphocyte-associated protein 4 (*CTLA-4*) inhibitors being the main options [78]. RCC treatment is moving towards personalized and combination therapies, especially in the treatment of advanced and metastatic RCC, where the combination of targeted drugs and immunotherapy has become a key option.

#### Expression and prognostic implications of MUC1 in RCC

In recent years, the relationship between *MUC1* and RCC has attracted the attention of researchers, and multiple studies have found that the expression of *MUC1* in RCC is closely related to the pathological type, grading, and prognosis of patients [79]. Immunohistochemical assays showed that *MUC1* can be detected on the cell membrane surface and in the cytoplasm. *MUC1* is primarily expressed at the apical portion of epithelial cells in the distal convoluted tubules, Henle’s loops, and collecting ducts of the kidneys, with no expression in the proximal convoluted tubules [80]. Fujita et al. [81] found that high expression of *MUC1* in RCC tumor tissues, with significantly higher expression in tissues from locally advanced, metastatic lesions, and higher tumor cell nuclear grading. Leroy et al. [82] also confirmed that *MUC1* shows heterogeneous and aggressive high expression in RCC tumor tissues. Immunohistochemical analysis suggested that *MUC1* stained throughout the cytomembrane and cytoplasm and that *MUC1* can be used as a indicator for predict the prognosis of pT1 RCC [83].

**Fig. 3:**
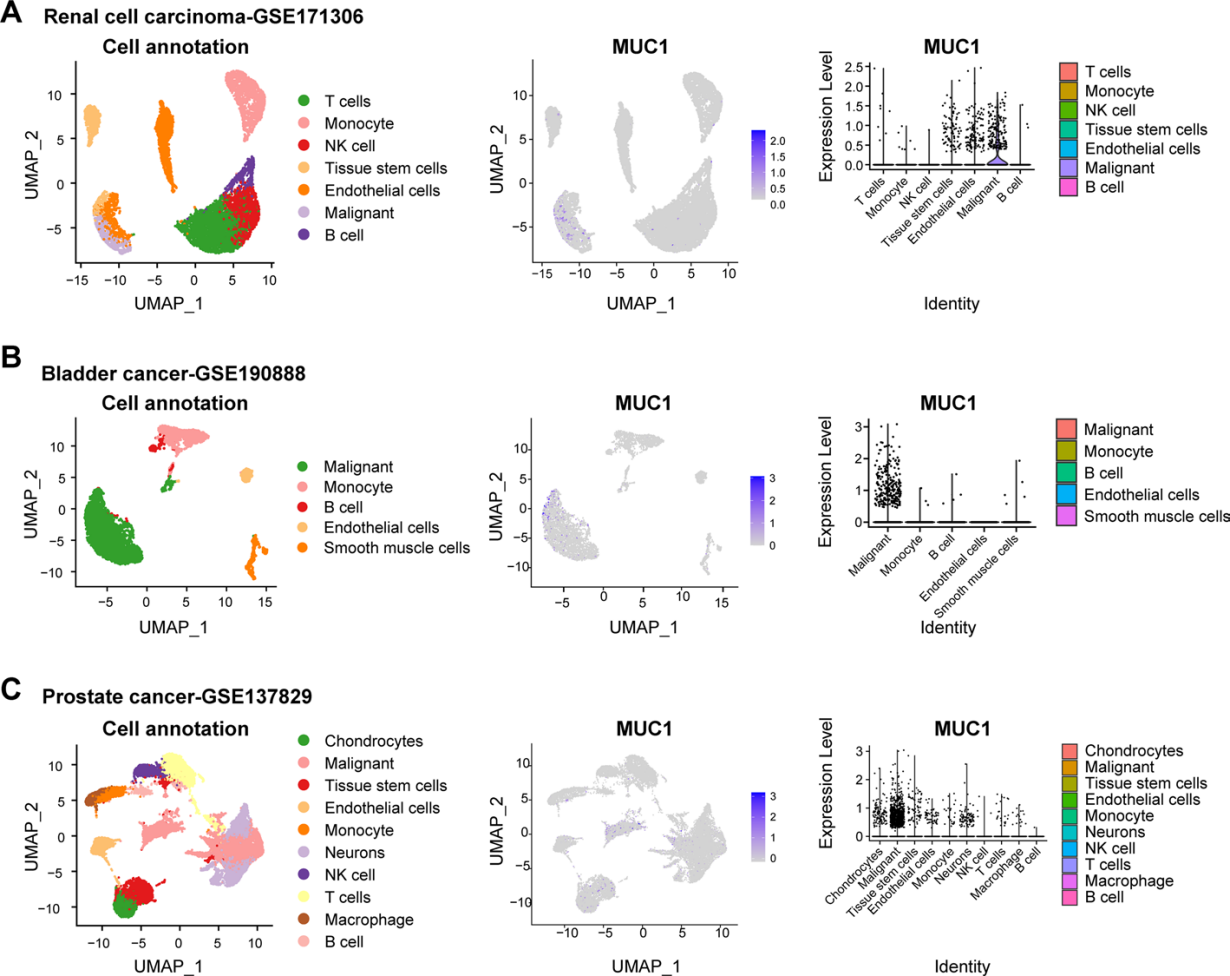
Single-cell RNA sequencing (scRNA-seq) analysis of *MUC1* expression in different cells of genitourinary cancers. **A** The scRNA-seq results of GSE171306 indicate that renal cell carcinoma tissue is mainly composed of T cells, monocytes, natural killer (NK) cells, tissue stem cells, endothelial cells, malignant cells, and B cells, with *MUC1* primarily expressed in malignant cells. **B** The scRNA-seq results of GSE190888 show that bladder cancer tissue is mainly composed of malignant cells, monocytes, B cells, endothelial cells, and smooth muscle cells, with *MUC1* primarily expressed in malignant cells. **C** The scRNA-seq results of GSE137829 reveal that prostate cancer tissue is mainly composed of chondrocytes, malignant cells, tissue stem cells, endothelial cells, monocyte, neurons, NK cells, T cells, macrophages, and B cells, with *MUC1* primarily expressed in malignant cells

Numerous clinical retrospective studies have also indicated a negative correlation between the expression of *MUC1* in primary RCC and patients’ overall survival (OS) and disease-free survival after surgery, and tumors with lymph node involvement or distant metastasis showed stronger *MUC1* expression than M0N0 stage tumors [84]. Kraus et al. [85] found that overexpression of *MUC1* in patients with RCC was statistically significant in tumor size, distant metastasis, and large vein invasion (*P* < 0.05), and that expression of neoglycosylation epitopes exposed by *MUC1* glycosylation insufficiency showed statistically significant differences in tumor recurrence, metastasis, and lethality (*P* < 0.001). Differences in *MUC1* expression can also be used to distinguish between type I and type II papillary renal cell carcinoma (PRCC) and as a marker for distinguishing multilocular cystic renal cell carcinoma (MCRCC) and renal cell carcinoma cystic change (RCC-CD) [86, 87]. In summary, *MUC1* can function as an independent prognostic marker for RCC, with high expression indicating poor prognosis. We also analyzed the expression of *MUC1* in the RCC single-cell RNA sequencing dataset GSE190888 and found that *MUC1* was significantly highly expressed in malignant cells (Fig. 3A).

#### Regulatory mechanism of MUC1 in RCC

**Fig. 4:**
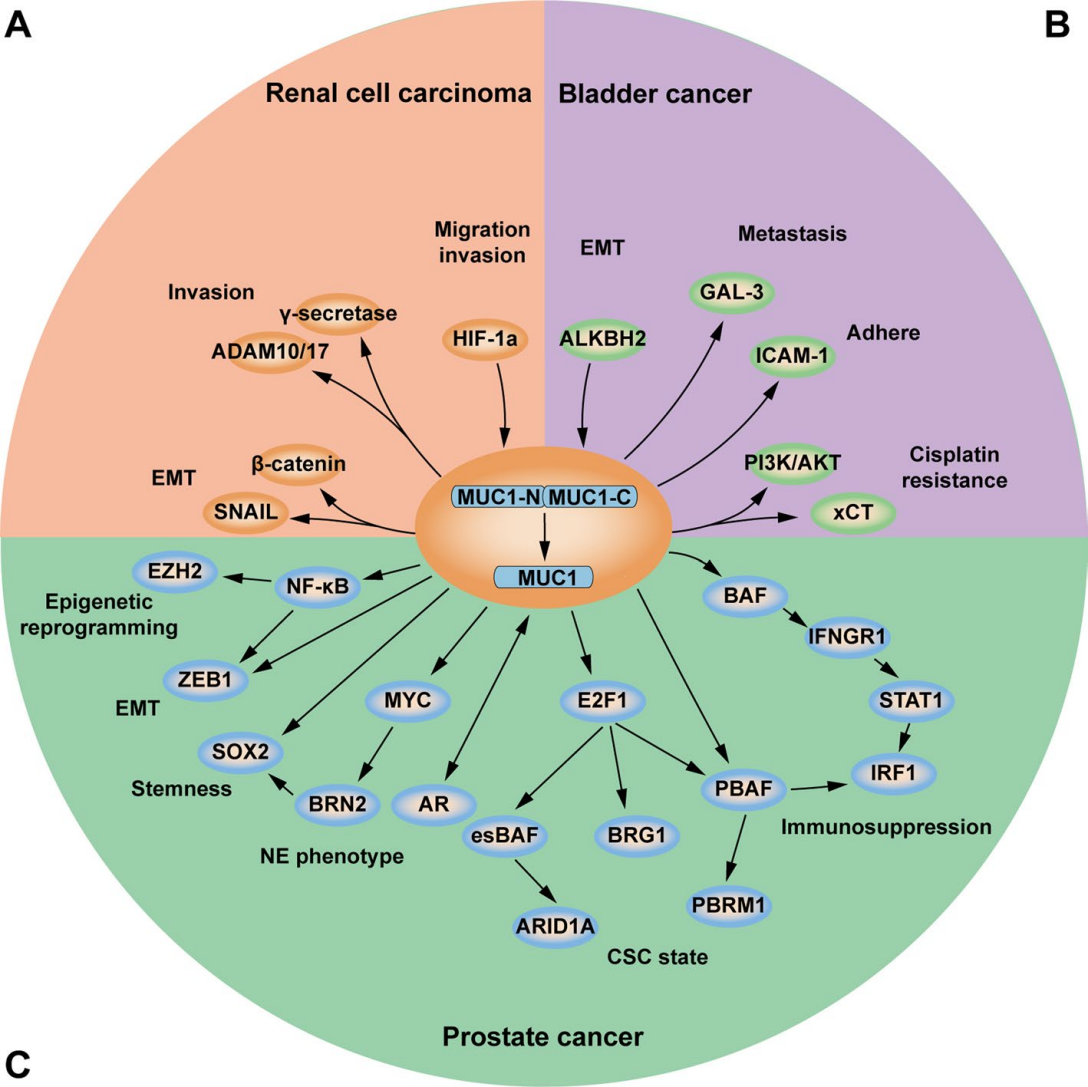
Regulatory mechanisms of *MUC1* in genitourinary cancers. **A** Proposed models of *MUC1* function in renal cell carcinoma epithelial–mesenchymal transition (EMT), migration, and invasion. **B** Proposed models of *MUC1* function in bladder cancer EMT, metastasis, adherence, and cisplatin resistance. **C** Proposed models of *MUC1* function in prostate cancer epigenetic reprogramming, EMT, stemness, NE phenotype, cancer stem cell (CSC) state, and immunosuppression

Existing studies indicate that *MUC1* is involved in RCC progression mainly through hypoxia, EMT, drug resistance, immune regulation, and metabolic reprogramming (Fig. 4A). Lucarelli et al. [88] found that clear cell renal cell carcinoma (ccRCC) expressing *MUC1* were characterized by metabolic reprogramming involving glucose and lipid metabolic pathways, and that inhibition of *MUC1* expression reduced cell viability and survival rates and enhanced sensitivity to cisplatin. They also discovered that *MUC1* expression can regulate the immune density of ccRCC by activating the classical pathway of the complement system and modulating immune infiltration, thus promoting the formation of an immune-silencing microenvironment [89]. Gnemmi et al. demonstrated that MUC1-C drives tumor cell EMT through the Wnt/β-catenin signaling pathway and interaction with the Snail promoter [90]. Studies have also found that MUC1-C nuclear localization drives invasiveness of RCC cells through a shedding enzyme (an aisintegrin and metalloproteinase)* ADAM10/17*)/γ-secretase-dependent pathway [91]. In Aubert’s research, it was found that *MUC1 *expression under hypoxic conditions was induced by a hypoxia inducible factor (HIF)-dependent mechanism and that *MUC1* was directly regulated by *HIF-1a* and affected the invasive and migratory properties of RCC cells [92]. Furthermore, Chen et al. [93] found that *MUC1* expression was increased in sunitinib-resistant RCC strains by exploring the gene expression omnibus (GEO) database, suggesting that *MUC1* may play a crucial role in RCC sunitinib resistance.

#### Targeting MUC1 in RCC therapy

In recent years, new discoveries on the structure and function of *MUC1* as well as the continuous development of research techniques have greatly propelled the development of research strategies targeting *MUC1* as a therapeutic target, and antitumor vaccines targeting *MUC1* have begun to be used in the treatment of advanced RCC [94]. Wierecky et al. [95] conducted a phase I clinical trial including 20 patients with metastatic RCC, and prepared dendritic cells (DC-MUC1-PADRE) with added T helper epitope (pan HLA-DR binding peptide PADRE). These cells exhibited high cytotoxicity and were able to kill RCC cells in an antigen-specific and HLA-A2 restricted manner, with objective remission being achieved in 3 patients and MUC1-specific cytotoxic T-lymphocyte responses being detected in the 11 patients [96]. Rittig et al. [97, 98] conducted a phase I/II clinical trial in 30 patients with metastatic RCC to evaluate the feasibility, safety, and immunological and clinical response of an mRNA vaccine (containing *MUC1*, carcinoembryonic antigen (*CEA*), Her2/neu, telomerase, survivin, and *MAGE-A1*) using adjuvant granulocyte–macrophage colony-stimulating factor (GM-CSF). The mRNA vaccine was found to generate CD8^+^ and CD4^+^ immune responses in patients, with 1 patient achieving partial remission and 15 achieving stable disease. Moreover, MVA-MUC1 is a modified Ankara vaccinia virus that expresses *MUC1*. Fend et al. [99] demonstrated that intravenously injected MVA-MUC1 can generate MUC1-specific CD8^+^ T cells that inhibit tumor growth in RCC, and that the anti-tumor efficacy was further enhanced when combined with a Toll-like receptor 9 (*TLR9*) agonist.

TG4010 is a cancer vaccine expressing human *MUC1* and *IL-2* genes (MVA-MUC1-IL-2) composed of a highly attenuated modified Ankara virus strain [100]. The safety profile of TG4010 has been confirmed in multiple phase I/II clinical trials in a variety of malignancies [101–103]. In metastatic RCC, TG4010 in combination with *IL-2* and *IFN-α2a* cytokines increased the specific immune response in CD4^+^ and CD8^+^ T cells and overall survival (OS) was significantly prolonged in the conjoined treatment group and the treatment was well-tolerated [104]. Hillman et al. [105] evaluated the combined use of tumor irradiation and TG4010 in a mouse tumor model. They found that preinjection of TG4010 before local tumor irradiation enhanced the immune responses against tumor antigens, resulting in specific antitumor immune effects. Moreover, ClinicalTrials shows two MUC1-related clinical trials in RCC (2024.05.20). One trial evaluating the treatment of advanced RCC with anti-CD3-MUC1 bispecific antibody was withdrawn, and another trial assessing P-MUC1-C-ALLO1 allogeneic CAR-T-cell therapy for advanced or metastatic RCC is in progress.

### MUC1 and BCa

BCa is a common malignant tumor of the male GU tract [106]. BCa is classified into non-muscle-invasive bladder cancer (NMIBC) and muscle-invasive bladder cancer (MIBC) based on the depth of tumor invasion. Depending on the stage of cancer, the patient’s physical condition, and other factors, treatment options for BCa include surgery, chemotherapy, immunotherapy, radiotherapy, and targeted therapy. Surgery is the main treatment for BCa [107]. For early-stage patients with BCa, transurethral resection of bladder tumor (TURBT) combined with intravesical chemotherapy is the primary treatment. For muscle-invasive bladder cancer, partial cystectomy and radical cystectomy can be performed [108]. Adjuvant and neoadjuvant chemotherapy are effective treatment options for MIBC or metastatic bladder cancer [109]. Immunotherapy is particularly suitable for patients with advanced BCa who are ineligible for chemotherapy or for those who do not respond to chemotherapy [110]. Targeted therapy has developed rapidly in recent years, especially for advanced or metastatic bladder cancer, including fibroblast growth factor receptor (*FGFR*) inhibitors and antibody–drug conjugates (ADCs) [111]. Additionally, radiotherapy can be used for MIBC patients who are inoperable or refuse surgery [112].

#### Expression and prognostic implications of MUC1 in BCa

In normal bladder tissue, *MUC1 *is expressed on the apical membrane of umbrella cells, forming a physical barrier along with other mucins [113]. This barrier plays a crucial role in preventing damage to epithelial cells caused by physicochemical factors (abnormal pH, osmolarity, and oxygen ion concentration in urine), preventing bacterial adhesion, inhibiting the nucleating and adhesive effects of calcium oxalate crystals [8]. Currently, *MUC1* is primarily utilized in histological and serological diagnostics of BCa. *MUC1* is present in the luminal surface of normal urothelial cells of the urinary bladder, exhibiting apical expression and polar distribution. However, the normal polar distribution of *MUC1* on the cell surface of bladder tumor cells disappeared, resulting in uniform surface expression and cytoplasmic expression [114]. Walsh et al. [115] examined the expression of *MUC1* in normal and BCa paraffin-embedded tissue sections, revealing weak expression in normal urothelial cells but 100% expression in BCa cells. Additionally, significant differences were observed in the expression patterns of tumors at different stages and grades, with high-grade and high-stage tumors displaying enhanced cytoplasmic staining. The expression of *MUC1* correlates positively with the staging and grading of BCa [116]. Through tissue analysis, Shigeta et al. [117] found that high MUC1-C expression was independently correlated with lower survival rate in patients with BCa and that MUC1-C expression was increased in cisplatin-resistant strains. Furthermore, MUC1 can also be used to differentiate invasive micropapillary carcinoma, papillary uroepithelial tumor of low malignant potential (PUNLMP), and low-grade papillary uroepithelial carcinoma (LGPUC) [118–121].

During malignant transformation, the MUC1-N can lose its polar distribution characteristics and enter the blood circulation. Therefore, *MUC1* could serve as a serum tumor marker. Simms et al. [122] detected serum *MUC1* levels in patients with different stages of BCa and in healthy individuals. The results showed that 47% of stage IV patients had serum levels above the normal range (*P* < 0.001), and stage III patients also exhibited higher *MUC1* levels than the control group, with a specificity of 97%. Serum *MUC1* levels are elevated in BCa patients, especially in advanced stages, making it a potential indicator for disease progression, prognosis, and recurrence prediction in advanced-stage tumors [8]. In addition, we analyzed the expression of *MUC1* in the BCa single-cell RNA sequencing (seRNA-seq) dataset GSE190888 and found that *MUC1* was significantly highly expressed in malignant cells (Fig. 3B).

#### Regulatory mechanism of MUC1 in BCa

*MUC1* can participate in BCa adhesion, metastasis, EMT and cisplatin resistance (Fig. 4B). Studies have revealed that MUC1-C can promote cisplatin resistance in BCa cells through two pathways: by activating the PI3K/AKT pathway to promotes the expression of ATP binding cassette subfamily B member 1(*ABCB1*), leading to the efflux of cisplatin from tumor cells, and by stabilizing x-cystine/glutamate transporter (xCT) protein expression, increasing intracellular levels of glutathione, resulting in reduced generation of reactive oxygen species (ROS) [117]. Sundar et al. conducted adhesion assays and atomic force microscopy (AFM) studies, confirming that *MUC1 *can bind to intercellular adhesion molecule 1 (*ICAM-1*), mediating the adhesion between BCa cells and endothelial cells [123]. Fujii et al. found that inhibition of AlkB Homolog 2 (*ALKBH2*) in BCa KU7 cell line decreased *MUC1* expression, induced G1-phase cell cycle arrest, increased E-cadherin and decreased vimentin expression, thereby inhibiting EMT in BCa tumor cells [124]. Suzuki et al. demonstrated that *GLA3* can bind to *MUC1* through poly-*N*-acetylgalactosamine, and *MUC1* carrying core 2 *O*-glycans serves as a molecular shield against NK cell attack, thereby promoting BCa metastasis [125].

#### Targeting MUC1 in BCa therapy

In order to accurately determine staging and facilitate specific targeted therapy of BCa, researchers envisioned a strategy of combining antibodies with radioisotopes. Monoclonal antibodies against *MUC1*, labeled with gamma-ray isotopes such as 99mTc and 111In, were locally injected into the bladder [126, 127]. While this approach could induce human anti-mouse antibody (HAMA) reactions, experiments have proven that intravenous administration of these complexes is safe and reliable. Furthermore, antibodies labeled with β-ray isotopes 67Cu and 188Re were utilized. It was found that ^67^Cu-labeled C595 monoclonal antibody could more effectively target highly malignant BCa cells, delivering lethal radiation doses to cancer cells while leaving adjacent normal tissues unaffected [128, 129]. The above experiments provided a foundation for the application of radioisotope-labeled antibodies. Hughes et al. utilized ^111^In-labeled C595 monoclonal antibody to detect primary, recurrent, invasive, and distant metastases in BCa, enabling improved clinical staging and assisting in the selection of patients suitable for radiotherapy [127]. The C595 monoclonal antibody labeled with ^188^Re possesses high immunoreactivity and specificity, and this complex can be localized specifically in BCa tissues [130].

Currently, clinical imaging techniques such as computed tomography (CT) and magnetic resonance imaging (MRI) are unable to detect early microscopic metastases and accurately stage pelvic lymph node metastases in BCa. Kunkler and colleagues administered intravesical anti-MUC1 monoclonal antibody (mAb) NCRC48 to 12 patients with radiologically confirmed BCa, demonstrating increased uptake of NCRC48 in cancer tissue compared to normal mucosa [131]. This suggests that MUC1 could be a valuable target antigen for monoclonal antibody therapy. Simms and others conducted precise staging of BCa using radioimmunoimaging techniques to guide surgical treatment plans. In this study, 21 BCa patients were intravenously injected with 99mTc-labeled C595 monoclonal antibody, and 16 of 20 patients with advanced BCa tested positive for the antibody at the tumor site, detecting three cases of pathologically confirmed pelvic lymph node metastasis not detected by preoperative CT scans [126]. No adverse reactions occurred in any of the patients, and there was no thyroid isotope accumulation. This indicates that the novel technology of 99mTc-labeled C595 monoclonal antibody can be used for staging diagnosis of BCa, especially in late-stage patients with lymph node and lung metastases. It can also be utilized for postoperative recurrence detection in BCa.

CV301 consists of recombinant poxvirus, modified vaccinia Ankara (MVA) and Fowlpox vaccine (FPV) encoding *CEA*, *MUC1*, and costimulatory molecules (*ICAM-1*, *LFA-3*, and *B7-1*) [132]. The efficacy of CV301 has been demonstrated in patients with a number of advanced solid tumors, including non-small cell lung cancer [132, 133]. A single-arm phase II clinical trial, involving 43 patients, evaluated the efficacy of CV301 in advanced BCa. Although the trial was terminated due to ineffectiveness, patients who benefited demonstrated T-cell responses against *CEA* and *MUC1* [134]. In addition, ClinicalTrials shows two completed clinical trials related to *MUC1* in BCa (20/05/2024). One assessed the effectiveness of PANVAC combined with BCG vaccine compared to BCG vaccine alone, and the other evaluated the efficacy of dendritic cell therapy expressing *MUC1* in recurrent BCa patients. PANVAC consists of a priming dose of recombinant vaccine vector and a booster dose of recombinant avian influenza vector, each encoding transgenes of *CEA* and *MUC1*, and three human costimulatory molecules (*B7-1*, *ICAM-1*, and *LFA3*). The results indicated that the OS period and time to adverse events were higher in the PANVAC combined with BCG vaccine group than in the BCG vaccine alone group.

### MUC1 and prostate cancer (PCa)

PCa is the most common malignant tumor in men [135]. The treatment options for prostate cancer depend on the stage of the disease, pathological features, the patient’s health condition, and treatment goals. For low-risk localized PCa, active surveillance is a recommended treatment strategy [136]. For patients with localized or locally advanced PCa, radical prostatectomy with lymph node dissection and radiotherapy are the main treatment methods [137]. For metastatic patients with PCa patients, androgen deprivation therapy (ADT) and chemotherapy are the primary treatments [138]. Targeted therapies, such as *PARP* inhibitors, have shown potential in PCa treatment in recent years, particularly for patients with specific gene mutations [139]. Additionally, immunotherapy has demonstrated some efficacy in castration-resistant PCa (CRPC), and for bone-metastatic castration-resistant PCa, radionuclide therapy can target areas where cancer cells have metastasized to the bones [140, 141]. For early-stage PCa, surgery and radiotherapy are the primary treatment options, while for advanced or metastatic PCa, hormone therapy, chemotherapy, immunotherapy, and targeted therapy are key approaches.

#### Expression and prognostic implications of MUC1 in PCa

Initially studied as a cancer-associated serum antigen (CASA) in PCa, the close relationship between *MUC1* and prostate cancer has gained increasing attention with further research. In normal prostate tissue, *MUC1* is expressed only on the glandular luminal surface of the epithelial cell membrane, similar to the expression in other normal glandular epithelial tissues [142]. In a study conducted by Kirschenbaum et al. [143] on 34 cases of PCa, *MUC1* positive expression was observed in 32 cases (94%), and most cases (62%) exhibited diffuse cytoplasmic distribution of *MUC1*, and the pattern of* MUC1* distribution was correlated with Gleason grade and clinical stage: cases with higher Gleason scores and advanced clinical stages showed predominantly diffuse expression. Studies have indicated that, overexpression of *MUC1* is significantly associated with tumor angiogenesis and adverse patient outcomes in PCa [144, 145].

Schut et al. [146] examined *MUC1* expression in benign prostatic hyperplasia, PCa, and PCa bone metastases tissues, and showed that all of these tissues expressed incompletely glycosylated *MUC1* epitopes. Additionally, the *MUC1* gene exhibited abnormal amplification in CRPC and neuroendocrine prostate cancer (NEPC). Compared with the 2% amplification rate in the cancer genome atlas (TCGA) primary PCa cohort, the amplification of *MUC1* significantly increased in the SU2C CRPC cohort (6%) and the NEPC-enriched CRPC cohort (30%) [147]. Lapointe and colleagues identified three subtypes of PCa based on differential gene expression profiles, in which *MUC1* was highly expressed and positively stained in subtypes II and III, which correlated with a high risk of invasiveness and recurrence of PCa (*P* = 0.003), suggesting that *MUC1* can serve as a molecular marker for the heterogeneity of PCa [148]. Moreover, the scRNA-seq analysis showed that *MUC1* was significantly highly expressed in malignant cells (Fig. 3C).

#### Regulatory mechanism of MUC1 in PCa

Studies have shown that *MUC1* can participate in epigenetic reprogramming, EMT, stemness, neuroendocrine (NE) phenotype, cancer stem cell (CSC) state and immunosuppression in PCa (Fig. 4C). Atobatele et al. [149] demonstrated that silencing transglutaminase-2 (*TG2*) using CRISPR–Cas9 can affect the transcriptional regulation of *MUC1* by inhibiting androgen receptor (*AR*) expression, leading to androgen insensitivity and malignancy in PCa cells. Studies have found that MUC1-C can directly bind to the MYC HLH/LZ structural domain to form the MUC1-C/MYC complex, which occupies the brain-2 (*BRN2*) promoter and induces the expression of *BRN2* and downstream *SOX2*, thereby regulating the NE phenotype of PCa cells [147]. Additionally, MUC1-C can directly bind to nuclear factor kappa B (*NF-κB*) p65, promoting the activation of NF-κB p65 target genes (enhancer of zeste homolog 2) *EZH2* and zinc finger e-box-binding homeobox 1 (*ZEB*1) to facilitate EMT and stemness as well as epigenetic reprogramming in PCa [150, 151].

MUC1-C is able to interact with E2F transcription factor 1 (*E2F1*) to induce the expression of esBAF subunit and activate the expression of notch receptor 1 (*NOTCH1*) and nanog homeobox (*NANOG*) genes to participate in CSC stemness of PCa cells [152, 153]. MUC1-C forms a nuclear complex with *E2F1*, activates the expression of esBAF, BRM/SWI2-related gene 1 (*BRG1*), and *PBAF* subunits, which in turn increases the expression of the target genes AT-rich interaction domain 1A (ARID1A) and polybromo 1 (*PBRM1*), and drives the expression of EMT, *NOTCH1*, *NANOG*, and OSK to participate in the CSC state of NEPC cells [154]. MUC1-C can activate the interferon gamma receptor 1 (*IFNGR1*) gene by forming a complex with *BAF*, upregulate the expression of* STAT1* and interferon regulatory factor 1 (*IRF1*), and directly regulate the expression of *IRF1* through *PBAF*, controlling the downstream expression of indoleamine 2,3-dioxygenase 1 (*IDO1*), tryptophanyl-TRNA synthetase (*WARS*), prostaglandin E synthase (*PTGES*), interferon-stimulated protein 15 (*ISG15*), and serpin family B member 9 (*SERPINB9*), thus participating in immunosuppression of PCa cells [155].

#### Targeting MUC1 in PCa therapy

Research on the application of MUC1-related tumor vaccines in PCa has been conducted, and TG4010 has been used in clinical trials for the treatment of PCa. Dreicer et al. [156] enrolled 40 PCa patients who had undergone surgery or radiation therapy and showed only an elevated PSA without evidence of recurrence. After subcutaneous injection of TG4010, the PSA doubling time (PSADT) significantly increased in 30% of the patients, and 53% of the patients exhibited MUC1-specific responses at baseline. Seven patients showed a MUC1-specific responses after vaccination, among whom six had PSADT higher than the average level. In a phase I trial of antigen-specific gene therapy for MUC1-positive advanced PCa patients using a recombinant vaccine virus encoding MUC1 and IL-2 (VV/MUC-1/IL-2), the results showed that VV/MUC-1/IL-2 enhanced the upregulation of IL-2 (*CD25*) and T-cell receptor (TcR), increased the CD4/CD8 ratio, enhanced T helper 1 type (TH1) cytokines (*INF-γ* and *TNF-α*) mRNA expression and induced NK cells activity and MUC1-specific cytotoxic T-cell activity independent of major histocompatibility complex (MHC) [157].

There are a number of MUC1-associated tumor vaccines have been proven effective in the treatment of metastatic CRPC (mCRPC). mCRPC is the state of prostate cancer that continues to progress and metastasize despite receiving ADT. Current treatment options for mCRPC include hormone therapy (enzalutamide, abiraterone, and apalutamide), chemotherapy (docetaxel and cabazitaxel), immunotherapy (sipuleucel-T), targeted therapy (*PARP* inhibitors), and radiopharmaceuticals. Bilusic et al. [158] conducted a phase I study involving 18 patients with mCRPC using a multitarget recombinant immunotherapy vaccine based on Ad5 PSA/MUC-1/brachyury. The results showed partial response in one patient, PSA levels reduction in five patients, and good tolerance to the vaccine in all patients with no treatment-related adverse events or dose-limiting toxicities (DLT). CV9104 is an mRNA vaccine encoding PSA, PSMA, PSCA, STEAP, PAP, and *MUC1* [159]. A randomized phase IIb study evaluated the safety and activity of CV9104 in chemotherapy-resistant patients with oligosymptomatic/asymptomatic mCRPC. No significant improvement in OS or progression-free survival (PFS) was observed after multiple vaccinations compared to placebo [160].

Additionally, for patients with CRPC who have developed resistance to chemotherapy, treatment options include hormonal targeted therapy, immunotherapy, targeted therapy, radionuclide therapy, and other emerging therapies. In a randomized phase IIa trial, 21 chemotherapy-resistant patients with CRPC received treatment with dendritic cells (DCs) loaded with tumor-associated antigens *NY-ESO-1*, *MAGE-C2*, and *MUC1*. The study demonstrated that immunotherapy using peripheral blood-derived DCs subpopulation is feasible and safe, inducing functional antigen-specific T cells [161]. Scheid et al. [162] conducted a phase I/II clinical trial encompassing 17 patients with non-mCRPC (nmCRPC), evaluating the safety of tumor MUC1 glycopeptide with Tn carbohydrates (Tn-MUC1). Eleven patients were found to have significantly improved PSADT (*P* = 0.037), and Tn-MUC1 could induce significant T-cell responses with five experiencing significant Tn-MUC1-specific intracellular cytokine responses in CD4^+^ and/or CD8^+^ T cells.

For prostate cancer patients who experience biochemical recurrence (BCR) after radical prostatectomy, treatment options are mainly based on the patient’s PSADT, pathological features, and overall health status. Available treatments include watchful waiting, adjuvant radiotherapy, hormone therapy, a combination of radiotherapy, and hormone therapy, as well as emerging targeted therapies and immunotherapy. L-BLP25 is a synthetic liposomal cancer vaccine targeting the extracellular tandem repeat sequence of the *MUC1* TAA [163]. An exploratory phase II study involving 16 male patients who underwent radical prostatectomy and experienced BCR showed that L-BLP25 vaccine could prolong PSADT and was suitable for hormone-naive patients with PCa with post-prostatectomy biochemical failure and low recurrence rates [164]. Furthermore, *MUC1* peptides can enhance its immunogenicity by combination with keyhole limpet hemocyanin (KLH) and certain immune adjuvants, leading to increased levels of IgG and IgM in vivo [165]. Dexamethasone administered in vivo to immune-deficient mice resulted in a significant up-regulation of the *MUC1 *expression levels in the tumor cells and a greater sensitivity to complement-mediated cytotoxicity, suggesting that dexamethasone can be used as an adjuvant for immune-targeted against *MUC1* therapy [166].

In addition, ClinicalTrials shows eight MUC1-related PCa clinical trials (2024.05.20). Among these six trials completed, one randomized multicenter phase II research trial evaluating two administration doses of TG4010 (MVA-MUC1-IL2) in patients with terminated PCa and one Ad-sig-hMUC-1/ecdCD40L vector vaccine for metastatic or recurrent PCa trial status unknown.

## Conclusion

*MUC1* acts as a dual role of tumor marker and immunotherapy target in GU cancers, although its specific functions are still challenging to define. Since the gene localization of *MUC1* is well defined, altering its expression in early cancers through gene regulation methods could potentially impact cell adhesion and polarity, thereby reducing the risk of cancer metastasis. With the development of antibody engineering allowing us to produce murine-derived single-chain antibodies and humanized antibodies. These molecules have very low immunogenicity in the human body, allowing for repeated intravenous administration without eliciting human anti-mouse antibody responses, and single-chain antibodies have better tissue permeability due to their smaller size, which makes them more effective for tumor-specific targeted therapies. In addition, the mRNA vaccine has a good application prospect due to its better safety, feasibility, and immunological and clinical responses, which will lay the foundation for the study of MUC1-targeted drugs and their clinical application in the treatment of GU cancers.

## Data Availability

The datasets used and analyzed during the current study are available from the corresponding author on reasonable request.

## Abbreviations

MUC1: Mucin 1
PTS: Proline, threonine, and serine
GU: Genitourinary
VNTRs: Variable number of tandem repeat sequences
PCa: Prostate cancer
BCa: Bladder cancer
RCC: Renal cell carcinoma
ED: Extracellular domai
TM: Transmembrane domain
CD: Cytoplasmic domain
MUC5B: Mucin 5B
SEA: Sea urchin spermatidin, enterokinase, and agarose
MUC1-N: MUC1 N-terminal
MUC1-C: MUC1 C-terminal
ER: Endoplasmic reticulum
EMT: Epithelial mesenchymal transition
Tyr: Tyrosine
GRB2: Growth factor receptor binding protein 2
GHR: Growth hormone receptor
IL-7R: Interleukin-7 receptor
IL-3R: Interleukin-3 receptor
MHC: Major histocompatibility complex
GSK3β: Glycogen synthase kinase 3β
ERK: Extracellular signal-regulated kinase
GAL3: Galectin-3
CTLs: Cytotoxic T lymphocytes
NK: Natural killer
TAA: Tumor-associated antigen
TKIs: Tyrosine kinase inhibitors
mTOR: Mammalian target of rapamycin
VEGF: Vascular endothelial growth factor
PD-1: Programmed cell death 1
PD-L1: Programmed death-ligand 1
CTLA-4: Cytotoxic T-lymphocyte associated protein 4
PRCC: Papillary renal cell carcinoma
MCRCC: Multilocular cystic renal cell carcinoma
RCC-CD: Renal cell carcinoma cystic change
ccRCC: Clear cell renal cell carcinoma
ADAM10/17: A aisintegrin and metalloproteinase 10/17
HIF: Hypoxia inducible factor
GEO: Gene expression omnibus
GM-CSF: Granulocyte–macrophage colony-stimulating factor
TLR9: Toll-like receptor 9
OS: Overall survival
NMIBC: Non-muscle-invasive bladder cancer
MIBC: Muscle-invasive bladder cancer
TURBT: Transurethral resection of bladder tumor
FGFR: Fibroblast growth factor receptor
ADCs: Antibody–drug conjugates
PUNLMP: Papillary uroepithelial tumor of low malignant potential
LGPUC: Low-grade papillary uroepithelial carcinoma
ABCB1: ATP binding cassette subfamily B member 1
xCT: X-cystine/glutamate transporter
ROS: Reactive oxygen species
AFM: Atomic force microscopy
ICAM-1: Intercellular adhesion molecule 1
ALKBH2: AlkB homolog 2
HAMA: Human anti-mouse antibody
CT: Computed tomography
MRI: Magnetic resonance imaging
mAb: Monoclonal antibody
MVA: Modified vaccinia Ankara
FPV: Fowlpox vaccine
CEA: Carcinoembryonic antigen
ADT: Androgen deprivation therapy
CASA: Cancer-associated serum antigen
CRPC: Castration-resistant prostate cancer
NEPC: Neuroendocrine prostate cancer
TCGA: The Cancer Genome Atlas
NE: Neuroendocrine
CSC: Cancer stem cell
TG2: Transglutaminase-2
AR: Androgen receptor
BRN2: Brain-2
NF-κB: Nuclear factor kappa B
EZH2: Enhancer of zeste homolog 2
ZEB1: Zinc finger e-box-binding homeobox 1
E2F1: E2F transcription factor 1
NOTCH1: Notch receptor 1
NANOG: Nanog homeobox
BRG1: BRM/SWI2-related gene 1
ARID1A: AT-rich interaction domain 1A
PBRM1: Polybromo 1
IFNGR1: Interferon gamma receptor 1
IRF1: Interferon regulatory factor 1
IDO1: Indoleamine 2,3-dioxygenase 1
WARS: Tryptophanyl-TRNA synthetase
PTGES: Prostaglandin E synthase
ISG15: Interferon-stimulated protein 15
SERPINB9: Serpin family B member 9
PSADT: PSA doubling time
TH1: Thelper 1 type
mCRPC: Metastatic castration-resistant PCa
DLT: Dose-limiting toxicities
PFS: Progression-free survival
DCs: Dendritic cells
BCR: Biochemical recurrence
KLH: Keyhole limpet hemocyanin
